# Community mental health centres in Slovenia: assessment of the situation and needs

**DOI:** 10.1192/j.eurpsy.2025.690

**Published:** 2025-08-26

**Authors:** N. Petkovšek, V. Švab, U. Juhart, R. Zupan

**Affiliations:** 1 Mental health center, Health Center Logatec, Logatec; 2 Mental health, National Institute for Public Health, Ljubljana, Slovenia

## Abstract

**Introduction:**

Mental health reform was accepted in Slovenia in 2018 to provide accessible community treatment and care regionally for 40-70000 population. The implementation took place mostly with establishment of community mental health centers (CMHC), which included arround 20 000 patients in 16 community centers for the adult population. These CMHCs provide triage, needs assessment and treatment plans. The National Mental Health Plan did not adress the development of rehabilitation services. The institutional costs of treatment and care were not reduced, as well as not the number of institutional beds.

**Objectives:**

To assess the development of CMCH’s in Slovenia on the grounds of the data from the CMHC Logatec.

**Methods:**

The data about 5761 patients included in the treatment process in one of the CMHCs were gathered regarding basic demographic data, diagnosis and treatment mode, including the definition of care coordination.

**Results:**

2654 patients were treated in the home environment. Medical nurses were coordinating treatment in the majority of these patients. Occupational therapist, social worker and psychiatrists were part of the team in this group. 816 patients in this group were diagnosed with dementia and had also somatic ilness. 1011 were diagnosed with schizoprenia or other psychotic disorders. 3107 patients were treated in the outpatient care, the majority of them by psychologists. Psychiatrists and social worker were included in these care coordinating teams. 1219 of them were diagnosed with depressive and 1001 anxiety disorders.

**Conclusions:**

Community care teams in Slovenia included around 20 000 patients in three years time. The majority of these, specifically in Logatec are diagnosed with schizophrenia, dementia, depressive and anxiety disorders. Immediate access to service is still granted, with exception of psychological treatment which does not reach everybody in need in time. Development of CMCH’s should be led by needs assesment on the ground of developing data. It’s already obvious that increase of the number of psychologists to improve psychological assistance to people with cognitive decline and for young people in severe but mostly manageable psychological crisis is needed.

**Disclosure of Interest:**

None Declared

